# Outer Membrane Protein F Is Involved in Biofilm Formation, Virulence and Antibiotic Resistance in *Cronobacter sakazakii*

**DOI:** 10.3390/microorganisms9112338

**Published:** 2021-11-11

**Authors:** Jianxin Gao, Zhonghui Han, Ping Li, Hongyan Zhang, Xinjun Du, Shuo Wang

**Affiliations:** 1 Key Laboratory of Animal Resistance Biology of Shandong Province, Key Laboratory of Food Nutrition and Safety, School of Life Science, Shandong Normal University, Jinan 250014, China; jxgao@sdnu.edu.cn (J.G.); zhanghongyan@sdnu.edu.cn (H.Z.); 2 School of Food Science and Engineering, Qilu University of Technology, Shandong Academy of Sciences, Jinan 250353, China; zhhan@qlu.edu.cn; 3 School of Food Science and Engineering, Tianjin University of Science and Technology, Tianjin 300457, China; zoelxx@tust.edu.cn; 4 Tianjin Key Laboratory of Food Science and Health, School of Medicine, Nankai University, Tianjin 300071, China

**Keywords:** *ompF*, *Cronobacter sakazakii*, LPS, biofilm formation, adhesion/invasion

## Abstract

In some Gram-negative bacteria, *ompF* encodes outer membrane protein F (OmpF), which is a cation-selective porin and is responsible for the passive transport of small molecules across the outer membrane. However, there are few reports about the functions of this gene in *Cronobacter sakazakii*. To investigate the role of *ompF* in detail, an *ompF* disruption strain (Δ*ompF*) and a complementation strain (cp*ompF*) were successfully obtained. We find that OmpF can affect the ability of biofilm formation in *C. sakazakii*. In addition, the variations in biofilm composition of *C. sakazakii* were examined using Raman spectroscopy analyses caused by knocking out *ompF*, and the result indicated that the levels of certain biofilm components, including lipopolysaccharide (LPS), were significantly decreased in the mutant (Δ*ompF*). Then, SDS-PAGE was used to further analyze the LPS content, and the result showed that the LPS levels were significantly reduced in the absence of *ompF*. Therefore, we conclude that OmpF affects biofilm formation in *C. sakazakii* by reducing the amount of LPS. Furthermore, the Δ*ompF* mutant showed decreased (2.7-fold) adhesion to and invasion of HCT-8 cells. In an antibiotic susceptibility analysis, the Δ*ompF* mutant showed significantly smaller inhibition zones than the WT, indicating that OmpF had a positive effect on the influx of antibiotics into the cells. In summary, *ompF* plays a positive regulatory role in the biofilm formation and adhesion/invasion, which is achieved by regulating the amount of LPS, but is a negative regulator of antibiotic resistance in *C. sakazakii*.

## 1. Introduction

*Cronobacter sakazakii* is an opportunistic food-borne pathogen that is associated with outbreaks of life-threatening bacteremia, meningitis and necrotizing enterocolitis (NEC) in neonates and infants, with case fatality rates reported to be as high as 40–80% and survivors frequently left with severe neurological and developmental disorders [1,2]. In addition, while not only causing newborn disease, *C. sakazaki* also infects adults. *C. sakazakii* CC4 and *C. sakazakii* ST12 have been recognized as specific pathovars associated with particular neonatal and adult infections [3,4]. In addition, *C. sakazakii* also has a wide range of habitats, and it has been found in powdered milk substitutes, meters and even in domestic kitchen sponges [5,6,7].

*C. sakazakii* strains have the capacity to invade and translocate through the Caco-2 and human brain microvascular endothelial cell lines [8]. These organisms can form biofilms, which can adhere to substrate surfaces, survive in the presence of antibiotics and disinfectants and enhance the resistance of cells to environmental stress [9]. In addition, Lehner and Kim reported that *Cronobacter* spp. have the ability to form biofilms to enhance adherence and improve pathogenesis [10,11]. Hartmann et al. suggested that the hypothetical proteins ESA_00281 and ESA_00282 have a strong impact on biofilm formation and contribute to the adhesion of *C. sakazakii* to Caco-2 intestinal epithelial cells [12]. In our previous work, using a random transposon insertion mutant library, we showed that the interactions of the *ompF* gene was mostly associated with biofilm formation in *C. sakazakii*. Therefore, it is reasonable to speculate that the *ompF* gene plays a vital role in the pathogenesis of *C. sakazakii*. However, there is very little information about how this gene works exactly on the biofilm-related pathogenesis of *C. sakazakii*.

Several outer membrane porin proteins (OMPs) have been discovered in Gram-negative bacteria; these proteins are β-barrel integral membrane proteins that form nonspecific water-filled channels, allowing the passive diffusion of ions and molecules with molecular masses up to 600 Da [13,14]. OmpF is one of the most abundant proteins found in the outer membranes (OMs) of Gram-negative bacteria [15,16]. Prehna et al. demonstrated that OmpF and outer membrane protein C (OmpC) not only function to import ions and protein toxins but also contribute to the export of YebF, a 10.8 kDa soluble endogenous protein, in *E. coli* [17]. In addition, Nicholas et al. reported that an intrinsically disordered protein could tunnel through OmpF to deliver an epitope signal to the cell and initiate cell death. Additionally, OmpF can also serve as an entryway into cells for many antibiotics [18,19]. However, the function of *ompF* in *C. sakazakii* is still unclear. In this study, we generated an *ompF* deletion mutant (Δ*ompF*) and complementation controls in *C. sakazakii* ATCC BAA-894 to investigate the function of this gene. The biofilm formation ability was estimated, and the differences in the biochemical components of the biofilms of the different *C. sakazakii* ATCC BAA-894 strains were analyzed. Meanwhile, the ability of the *C. sakazakii* strains to invade or adhere to HCT-8 cells was investigated by an invasion/adhesion assay. In addition, we studied the difference in cell permeability and antibiotic resistance between the *C. sakazakii* ATCC BAA-894 wild_type and mutant strains. The research aim was to demonstrate the role of the *ompF* homolog of *C. sakazakii* ATCC BAA-894 in virulence and permeability.

## 2. Materials and Methods

### 2.1. Bacterial Strains, Plasmids and Culture Conditions

All the bacterial strains and plasmids used in this study are listed in Table S1. *Cronobacter sakazakii* and *Escherichia coli* were incubated on Luria–Bertani (LB; Difco, MD, USA) at 37 °C with constant shaking at 200 rpm. When needed, kanamycin, chloramphenicol or ampicillin was used at final concentrations of 100 µg/mL, 10 µg/mL and 100 µg/mL, respectively.

### 2.2. Construction of ompF Deletion Mutant

We constructed an *ompF* mutant using the Lambda-Red recombination system according to procedures reported by Kim et al. [20]. Briefly, using pET-26b plasmid as a template and KF/KR as primers, the kanamycin resistance cassette was amplified by PCR. Then, after digesting with *Bam*HI and *Sal*I, the PCR product was cloned into the pMD18-T vector to generate pMDK. The upstream and downstream flanking regions of the *ompF* gene were amplified with the two primer pairs 413UF/413UR and 413DF/413DR using the genomic DNA of *C. sakazakii* ATCC BAA-894 as template; the whole-genome sequence was obtained from GenBank. Then, the upstream DNA fragment of *ompF* was digested with *KpnI* and *Bam*HI and inserted into the corresponding sites of pMDK to yield pMDKU. Then, both the downstream DNA fragment of *ompF* and the pMDKU plasmid were digested with *Sal*I and *Hin*dIII and then ligated by T4 DNA ligase to generate the plasmid pMDKUD, which was then transformed into *E. coli* DH5α. The *ompF*-upstream–kana–*ompF*-downstream product double digested with restriction endonucleases was transformed via electroporation into *C. sakazakii* ATCC BAA-894 (the wild_type, WT) harboring the pKD46 plasmid. Kanamycin-resistant transformants were selected to further verify successful mutation (Δ*ompF*). The sequences of all primers used are listed in Table S2.

### 2.3. Complementation Study

The complement plasmid pACYC184-*ompF*, which contains the *ompF* gene sequence and the native promoter, was constructed. The *ompF* sequence was amplified from wild_type *C. sakazakii* ATCC BAA-894 genomic DNA using the primer pair *ompF*-pACYC-F/*ompF*-pACYC-R (restriction enzyme sites *Bam*HI and *Sal*I were introduced into the primers). The PCR product was digested with the restriction endonucleases *Bam*HI and *Sal*I and cloned into the pACYC184 plasmid. Subsequently, the recombinant plasmid (pACYC184-ompF) was transferred into the Δ*ompF* mutant to generate the *ompF* complementation strain (cp*ompF*) [21]. Nucleotide sequencing was performed to confirm that the *ompF* coding region was in the pACYC184-*ompF*.

### 2.4. Growth Curves

The growth curve was interpreted by measuring the concentration of bacteria at different times by an ultraviolet spectrophotometer. The *C. sakazakii* strains ATCC BAA-894, Δ*ompF* and cp*ompF* inocula were cultured at 37 °C overnight without shaking in LB solid medium, and then subcultured in 50 mL of LB liquid medium at a ratio of 1:100. The cultures were incubated at 37 °C for 14 h with shaking at 200 rpm. The optical density at a wavelength of 600 nm (OD_600_) was measured every hour. Samples with excessive concentrations (OD_600_ > 0.8) were diluted before measurement and the final OD_600_ value was calculated by multiplying the dilution factor by the OD_600_ value of the diluted bacterial solution.

### 2.5. Morphological Differences

Scanning electron microscopy (SEM, Hitachi, Japan) was employed to observe the morphological differences among WT, Δ*ompF* and cp*ompF* strains. All the strains were collected by centrifugation in the logarithmic phase of growth. Following washing with phosphate-buffered saline (0.1 mmol/L) 2 times, the strains of BAA-894 (wild type), mutant and complementation were fixed with glutaraldehyde (2.5%, wt/vol) at 4 °C for 24 h, respectively. Then, the cells were rinsed with distilled water 3 times and fixed with osmium tetroxide (1%) for 1 h, which was followed by dehydration for 10 min with a series of alcohol (25–100%). The bacteria were further freeze-dried under vacuum for 4 h. Finally, SEM was used to examine the dehydrated bacterial powder using an accelerating voltage of 5 kV.

### 2.6. Analysis of Biofilm Formation Ability

The ability of biofilm formation was performed with cultures grown in 96-well polystyrene plates using crystal violet staining according to the method developed by Hu Lan with some modifications [22]. The *C. sakazakii* strains were inoculated overnight on LB at 37 °C with shaking (200 rpm). Then, the strains were transferred into 7 mL fresh medium (1:100 dilution) and grown until the cells reached the logarithmic period. Two hundred-microliter aliquots were added in triplicate into 96-well plates, and the plates were incubated at 37 °C for 48 h without shaking. The plates were gently rinsed 3 times with sterile PBS, and the adherent bacterial cells were fixed with 200 µL of 99% methanol for 15 min. Then, the plates were air-dried and stained with 200 µL of 0.1% crystal violet (CV) for 30 min at room temperature. After rinsing 3 times with distilled water, the CV bound to the biofilm was released with 200 µL of 95% ethanol for 20 min. The absorbance was determined at 570 nm by a Sunrise Basic microplate reader (Tecan, Austria). The biofilm assay was performed in three separate experiments for each strain.

### 2.7. Determination of Biofilm Biochemical Components

Biofilm biochemical components in *Cronobacter sakazakii* were determined by Raman spectroscopy analyses and the same methods as in our previous work [23].

### 2.8. Analysis of C. sakazakii LPS by SDS-Polyacrylamide Gel Electrophoresis (SDS-PAGE)

To investigate the function of *ompF* in *C. sakazakii* ATCC BAA-894 (WT), Δ*ompF* and cp*ompF* strains, the LPS was extracted from strains WT, Δ*ompF* and cp*ompF*, using the modified hot phenol–water method reported by Hong et al. [24]. Briefly, WT, ∆*ompF* and cp*ompF* strains were cultured overnight on LB medium at 37 °C and subcultured as a 1% overnight culture in 100 mL of LB medium. After the strain was cultured to 10^8^ CFU/mL, 100 mL of each cell suspension was added to an equal volume of 45% phenol solution for 5 min, and then an equal volume of 95% phenol solution was added with vigorous stirring for 20–30 min at 68 °C. When the solution cooled to about 10 °C, it was centrifuged at 5000× *g* for 45 min at 10 °C. The LPS was fractionated in the upper aqueous phase, and residual phenol was removed by dialyzing against water to obtain crude LPS. The LPS was further extracted with DNase I, RNaseA and proteinase K to eliminate contaminants. The purified LPS was finally precipitated with acetone. The LPS samples were subjected by SDS-polyacrylamide gel electrophoresis (SDS-PAGE) using a 5% (wt/vol) stacking gel and a 15% (wt/vol) resolving gel, which was followed by silver staining [25]. The images were visualized and photographed with a Molecular Imager Gel Doc^TM^ XR^+^ (Bio-Rad Laboratories, Inc., Hercules, CA, USA).

### 2.9. Adhesion/Invasion Assay

An invasion assay on the *C. sakazakii* strain was conducted following a modified method of Rogers et al. to determine the adhesion/invasion of the bacteria [26]. HCT-8 cells (ATCC CCL-244, Manassas, Virginia) were cultured in RPMI 1640 (Invitrogen, Carlsbad, CA, USA) containing 10% (vol/vol) fetal bovine serum (FBS, Invitrogen, Waltham, MA, USA) at 37 °C and 5% CO_2_. *C. sakazakii* strains were grown overnight at 37 °C in aerobic conditions, and then overnight cultures of *C. sakazakii* were transferred into fresh LB medium. The *C. sakazakii* cells were harvested by centrifugation (at 3000× *g* for 5 min at 4 °C) and then washed twice and resuspended with RPMI 1640. For the invasion assays, the *C. sakazakii* cells in RPMI 1640 were added onto washed HCT-8 cells that were >90% confluent (approximately 1 × 10^8^ CFU/well) in 6-well tissue culture plates, giving a multiplicity of infection (MOI) of 100. After incubating for 3 h in the presence of 5% CO_2_, the tissue culture plates were gently washed 3 times with PBS to remove nonattached bacteria. One milliliter of 0.1% Triton X-100 was added to each well to lyse the cells for 10 min. Finally, dilutions were plated onto plate count agar (PCA) to enumerate the CFU. All experiments shown were performed at least 3 times with a minimum of duplicate wells in each experiment.

### 2.10. Cell Permeability Assay

The *C. sakazakii* ATCC BAA-894 (wild type), Δ*ompF* and cp*ompF* strains in the log phase of growth were centrifuged at 3000× *g* for 10 min and incubated for 3 h in PBS containing 100 µg/mL arginine or lysine. The strains were collected by centrifugation at 3000× *g* for 10 min and then washed 3 times with PBS and resuspended with 1 mL of PBS. The cells were lysed using an ultrasonic cell disrupter (Scientz, Beijing, China) and centrifuged at 13,000× *g* for 10 min to obtain the supernatant. Then, the supernatant was derivatized with an AccQ·TagTM Chemistry Kit. Finally, the arginine and lysine were analyzed according to our previously established method [27].

### 2.11. Antimicrobial Susceptibility Testing

Antimicrobial susceptibility was tested using the standardized Bauer–Kirby agar disc diffusion method using Mueller–Hinton agar (Oxoid, CM0337, Basingstoke, Hampshire, UK) and following the instructions of the Clinical Laboratory Standards Institute (CLSI, 2015). *E. coli* ATCC 25,922 was used as a positive control. Discs of 6 antibiotics recommended for Enterobacteriaceae were tested, namely, gentamicin (10 mg), ampicillin (50 mg), penicillin (50 mg), tetracycline (30 mg), ciprofloxacin (5 mg) and kanamycin (50 mg) (Bio-Rad Laboratories, Marnes-la-Coquette, France). 

### 2.12. Statistical Analysis 

Data were analyzed using SPSS 19.0 (SPSS Inc., Chicago, IL, USA). The significant differences of the results were assessed by Student’s unpaired *t*-test and one-way analysis of variance (ANOVA) [28]. A *P* value of 0.05 was considered statistically significant. The data were presented as means ± deviations, and each experiment was performed in three independent replicate trials.

## 3. Results

### 3.1. Verification and Growth Characterization of the ompF Mutant 

**Figure 1:**
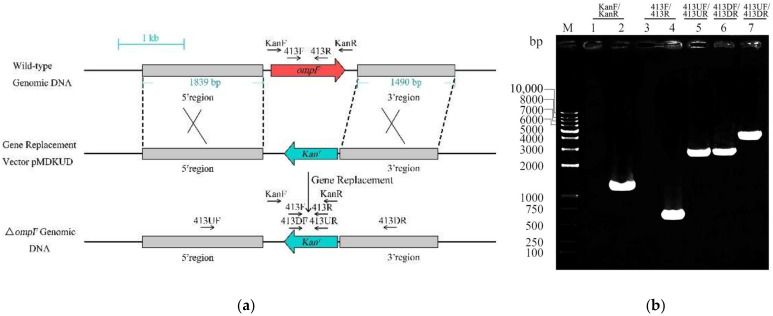
Construction and verification of the *ompF* deletion mutant. (**a**) Schematic representation of the ∆*ompF* construction via homologous recombination. (**b**) Verification of *ompF* homologous recombination events. Lane M, DL10000 marker; lanes 1 and 2, amplified with KF/KR, WT and Δ*ompF* as templates, respectively; lanes 3 and 4, amplified with 413F/413R, Δ*ompF* and WT as templates, respectively; lane 5, amplified with 413UF/413UR, Δ*ompF* as template; lane 6, amplified with 413DF/413DR, Δ*ompF* as template; lane 7, amplified with 413UF/413DR, Δ*ompF* as template.

**Figure 2:**
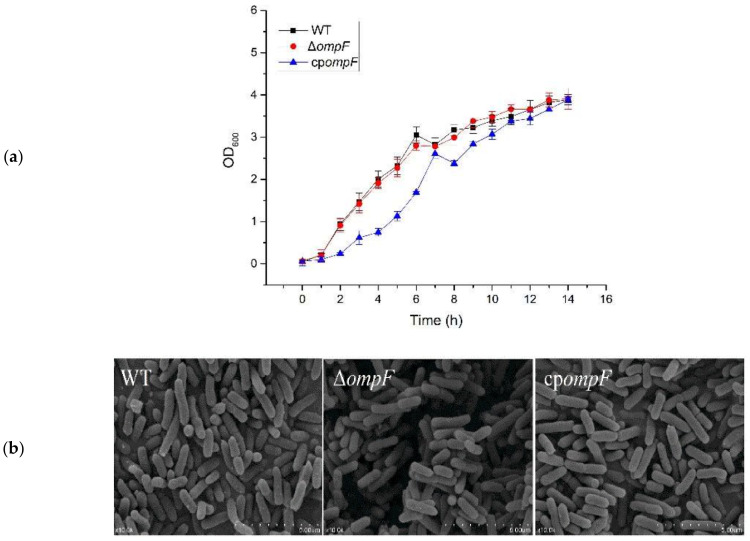
Growth and cell morphology of *C. sakazakii* in LB medium. (**a**) Growth of *C. sakazakii* in LB medium. Error bars represent the standard deviations from independent experiments performed in triplicate. (**b**) SEM to observe the cell morphology of WT, Δ*ompF* and cp*ompF.* Panels: WT, *C. sakazakii* ATCC BAA-894; Δ*ompF*, mutant strain; cp*ompF*, complementation strain.

The gene (ESA_02413) homologous to the *ompF* gene is located in the genome of *C. sakazakii* ATCC BAA-894. The gene is highly similar to the protein sequence of the *ompF* gene of *Salmonella enterica* (92%) and *Enterobacter cloacae* (80%). In order to investigate the functions of *ompF* in *C. sakazakii* ATCC BAA-894 pathogenesis, the *ompF* mutant (Δ*ompF*) was constructed by the Lambda-Red recombination technique. The *ompF* gene was replaced by the kanamycin resistance gene in the Δ*ompF* mutant (Figure 1a), which was confirmed by PCR (Figure 1b) and nucleotide sequencing. Then, we measured the growth curves to observe the effect of *ompF* on bacterial growth rate. Compared with the wild strain, the growth rate of the mutant strain did not show much difference, while that of the complement strain was slightly lower than that of the wild type in the first 6 h, but the difference was not significant (Figure 2a). The morphological characteristics of the wild_type, Δ*ompF* and cp*ompF* strains were examined by SEM, and the results showed that the three strains exhibited similar morphologies (Figure 2b). These results indicated that knocking out the *ompF* gene has no effect on the growth and morphology of *C. sakazakii* in LB medium.

### 3.2. Estimation of the Ability to Form Biofilms

**Figure 3:**
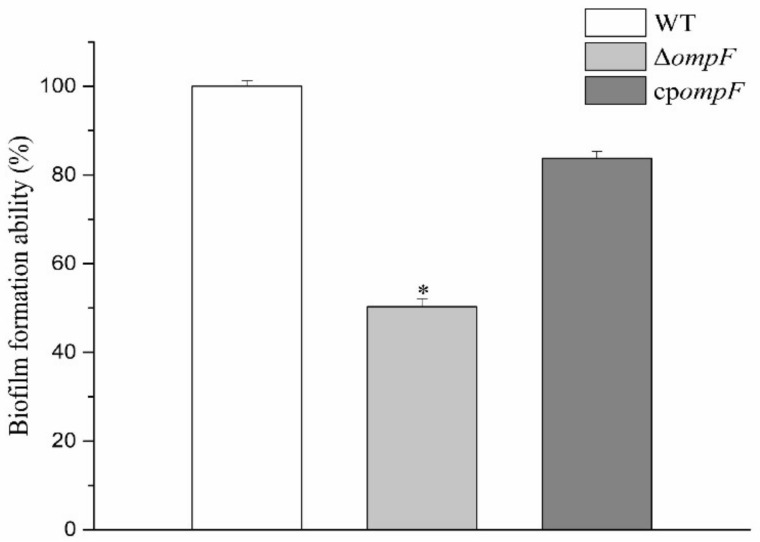
Comparison of the biofilm formation ability of different *C. sakazakii* isolates. The asterisks indicate that the percent biofilm formation by the mutant was significantly different (*p* < 0.05) from that by the wild_type strain.

A crystal violet staining assay was performed to study the influence of the *ompF* gene on the biofilm formation ability of *C. sakazakii*. Percentages of biofilm formation for the mutant and complementation strains relative to the WT strain are shown in Figure 3. The ability of biofilm formation in Δ*ompF* was significantly less (1.99-fold) than that by WT, while the complement strain showed similar biofilm formation ability to WT. These results indicated that the *ompF* gene played an important role in biofilm formation.

### 3.3. Differences in Biofilm Composition Examined by Raman Spectroscopy

**Figure 4:**
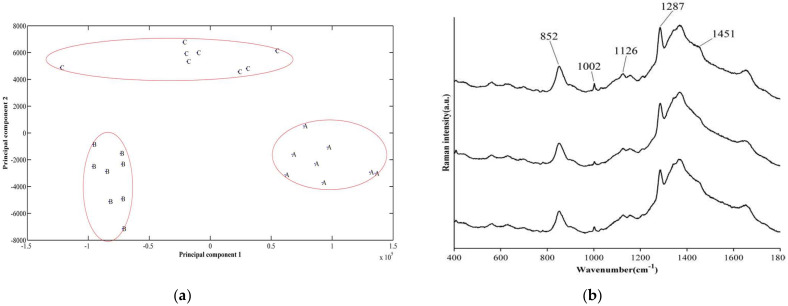
Comparison of the spectral features of *C. sakazakii* biofilms using Raman spectroscopy. (**a**) Principal component analysis of biofilm composition in *C. sakazakii* WT, Δ*ompF* and cp*ompF* strains. (**b**) Comparison of the spectral features of *C. sakazakii* biofilms using Raman spectroscopy. (A): complement strains, (B): mutant, (C): wild_type.

In order to explore which components of the biofilm changed after the *ompF* knockout, Raman spectroscopy was performed to further analyze the composition of the *C. sakazakii* biofilm. Additionally, the difference between biofilms formed by WT, Δ*ompF* and cp*ompF* was differentiated by constructing a two-dimensional principal component analysis (PCA) model. There were distinct differences among the Raman peaks representing the biofilm component by the wild_type, mutant and complementation strains (Figure 4a), suggesting that the biochemical components of the three strains are significantly different.

Raman spectra were plotted using MATLAB. A comparison of the Raman spectra suggested that the bands at 852, 1002, 1126, 1287 and 1451 cm^−1^ in Δ*ompF* were lower than those in the WT strain (Figure 4b). Peak assignment was carried out according to previously reported methods [29]. The band characteristic for the common bacterial polysaccharide-(1→ 3),(1→ 6)-α-d-glucan is located in 840–60 cm^−1^, and the bands at 852 cm^−1^ reflected glycogen [30,31]. Spectra of saccharides are characterized by groups of bands in the regions 1000–1200 cm^−1^ and 1002 cm^−1^ reflected β-D-glucose [32], respectively. The band at a wave number of 1126 cm^−1^ was from the skeletal υ(C-C) of the acyl backbones in lipid and disaccharides [30]. The 1287 cm^−1^ band was assigned to the characteristics of phosphodiester groups in nucleic acids, and the 1451 cm^−1^ band reflected fucose and galactosamine [30,32]. These results indicated that the absence of *ompF* negatively affects the content of some saccharides and lipids in biofilms.

### 3.4. Analysis of the LPS Content of the ompF Mutant and WT Strains

**Figure 5:**
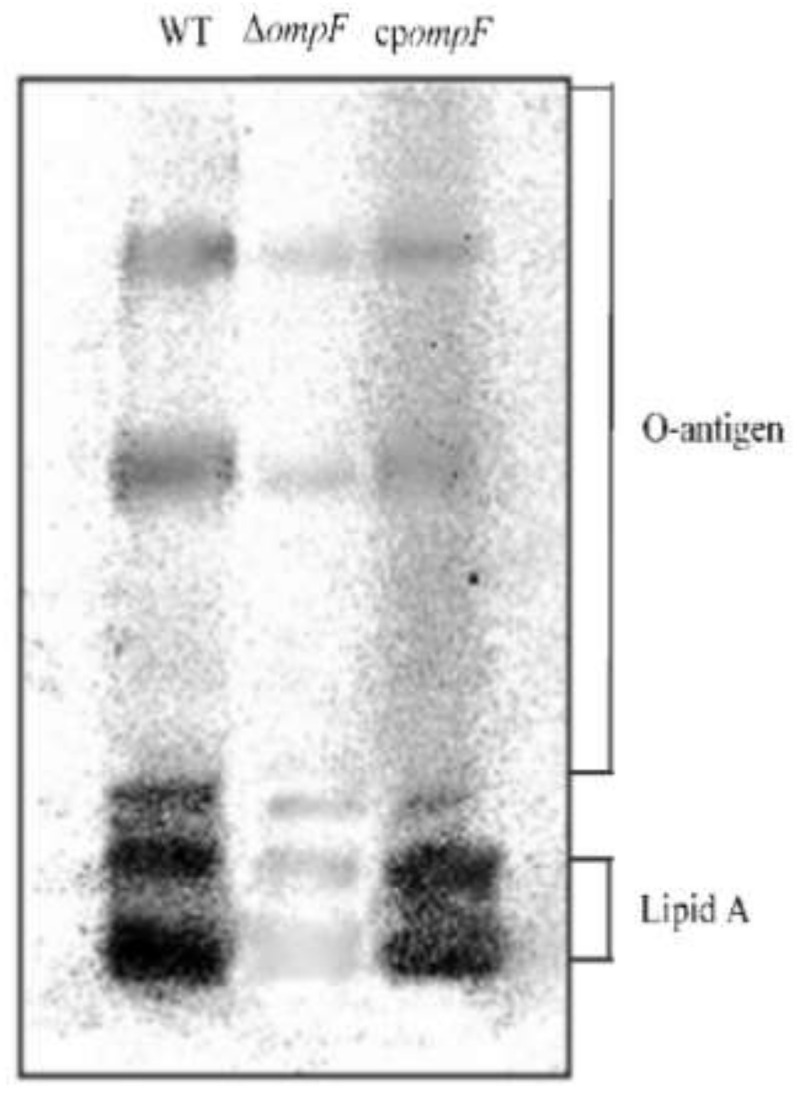
SDS-PAGE analysis of LPS extracted from *C. sakazakii* WT, Δ*ompF* and cp*ompF* strains.

Silver-stained LPS samples isolated from *C. sakazakii* ATCC BAA-894 wild_type, Δ*ompF* and cp*ompF* were analyzed by SDS-PAGE. The LPS profile of Δ*ompF*, including lipid A-core and O-antigen, had a lower molecular weight in contrast to that of the wild type, while the LPS profile of the complement strain was similar to that of WT (Figure 5). From the results, we concluded that *ompF* positively affects the LPS content in *C. sakazakii*.

### 3.5. ompF Affects Adhesion/Invasion

**Figure 6:**
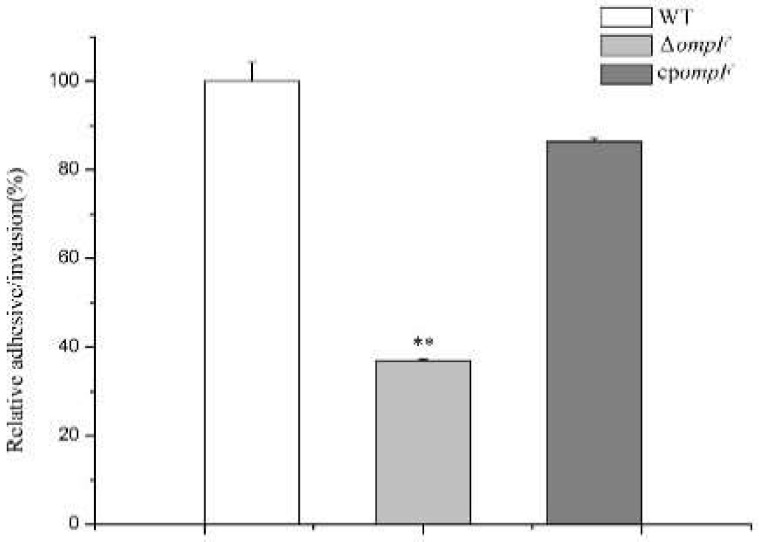
Adhesion to or invasion of epithelial cells by different *C. sakazakii* isolates. The asterisks indicate that the percent invasion by the mutant was significantly different (*p* < 0.01) from that by the wild_type strain.

The ability of adhesion to and invasion of tissue cells is considered to be essential in most pathogenic bacteria. An adhesion/invasion assay was conducted to determine the virulence-related functions of the *ompF* gene in *C. sakazakii*. The Δ*ompF* mutant showed significantly decreased (2.7-fold less than that of the WT) adhesion to and invasion of HCT-8 cells (Figure 6). However, the invasion of HCT-8 cells by the complement strain was similar to that by WT (Figure 6). The results suggested that *ompF* is a positive factor in the process of adhesion to and invasion of host cells by *C. sakazakii*.

### 3.6. Evaluation of Cell Permeability

As OmpF is a major outer membrane porin that controls the nonspecific diffusion of hydrophilic solutes in *C. sakazakii*, the permeability of the cell membranes of the *C. sakazakii* ATCC BAA-894 wild_type, Δ*ompF* and cp*ompF* was analyzed by measuring the concentration of arginine and lysine. As shown in Table 1, the arginine and lysine concentration of Δ*ompF* was significantly lower than that of the WT. Arginine was not detected in Δ*ompF*, and the concentrations observed in the complement strain were similar to those seen in the WT strain, indicating that the deletion of *ompF* substantially decreased the permeability of the cell membranes in *C. sakazakii*.

### 3.7. Estimation of Antibiotic Resistance

**Figure 7:**
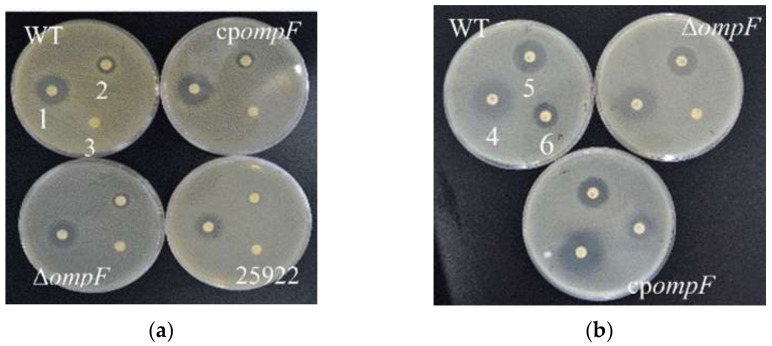
Antibiotic resistance of the *C. sakazakii* WT, Δ*ompF* and cp*ompF* strains. (**a**) 1, 2, 3 and the corresponding positions on the other plates represent gentamicin, ampicillin and penicillin, respectively; (**b**) 4, 5, 6 and the corresponding positions on the other plates represent tetracycline, ciprofloxacin and kanamycin, respectively.

In order to study the role of the *ompF* gene in the antibiotic resistance of *C. sakazakii*, antimicrobial susceptibility testing was carried out. Based on the size of the inhibition zone, we studied the resistance of the WT, Δ*ompF* and cp*ompF* to five small-molecule antibiotics (gentamicin, ampicillin, penicillin, tetracycline and ciprofloxacin). For all four antibiotics, the mutant showed significantly smaller inhibition zones compared to the WT, and the inhibition zones of the complementation strain were similar to those of the WT strain (Figure 7, Table 2). These results suggested that the *ompF* gene encodes a negative effector of antibiotic resistance.

## 4. Discussion

Biofilms composed of various major biological macromolecules are aggregates of microorganisms which act as a defense barrier and an important adhesive foundation in biofilm cells [33,34]. In our study, we found that the ability of biofilm formation decreased significantly in the *ompF*-deleted strain, suggesting the involvement of the *ompF* gene in biofilm formation. In addition, the content of some saccharides (glycogen, glucosamine, fucose and galactosamine) and lipids (lipid and phosphodiester groups in nucleic acids) that are constituents of LPS was dramatically higher in WT than that in Δ*ompF*, demonstrating that *ompF* is positively associated with LPS biosynthesis or the binding of LPS to bacterial surfaces. As an important component of biofilms, LPS has been found to play a vital role in biofilm formation in many Gram-negative bacteria [35,36,37]. In *Actinobacillus pleuropneumoniae* (*A. pleuropneumoniae*), the absence of the LPS O-antigen leads to a decrease in biofilm formation [38]. LPS is an amphipathic molecule that consists of hydrophobic lipid A, inner and outer oligosaccharide cores and O-antigen-specific polysaccharides [39]. The inner core region of LPS is important for outer membrane stability [40]. In *Escherichia coli* K-12, the major pore protein, OmpF, which is assembled as a trimer in the membrane, is tightly bound to the lipopolysaccharide [41]. In addition, Rouslan et al. have reported that the geometry and electrostatics of the OmpF surface make this protein a suitable binding site for LPS in *E. coli* [42]. Consistent with this finding, we speculate that the absence of OmpF in the *ompF* mutant of *C. sakazakii* renders LPS unable to adhere well to the outer membrane. This hypothesis was further confirmed by the SDS-PAGE analysis in this study. We compared the LPS content of the *C. sakazakii* WT and Δ*ompF* strains by SDS-PAGE and observed that the LPS content in the wild_type strain was dramatically higher than that in Δ*ompF*. According to our results, deletion of *ompF* affects the binding of LPS, the main component of biofilms, to the cell membrane, thereby weakening the ability of *C. sakazakii* to form biofilms.

Acting as a defense barrier and an important adhesive foundation in biofilm cells, biofilms are generally defined as an assemblage of microbial cells that adhere to a zoetic or abiotic surface to protect embedded cells against detachment due to flow shear [43]. Many studies have demonstrated that biofilms play an important role in the adherence to and invasion of human epithelial cells by pathogenic bacteria. Byrd et al. found that the biofilm polysaccharide Psl, as an adhesion-associated molecule, is required for adhesion of the bacteria to A549 epithelial cells [44]. In addition, Kunyanee et al. have reported the role of biofilm in the initial attachment and invasion of biofilm-related phenotypes of *B. pseudomallei* in the cellular pathogenesis of human lung epithelial cells [45]. Based on the result that the *C. sakazakii ompF* mutant showed a decreased biofilm formation phenotype, we hypothesized that this gene might be associated with the pathogenicity of *C. sakazakii*. This hypothesis was confirmed by the adhesion/invasion assay. Compared to the parent strain, the ability to adhere to and invade HCT-8 cells was dramatically decreased in the mutant, indicating that *ompF* may be a positive factor in the adhesion to and invasion of tissue cells by *C. sakazakii*. Therefore, *ompF* may regulate adhesion/invasion by affecting biofilm synthesis.

OmpF is one of the most important outer membrane proteins, which provide selective permeability, allowing nutrient molecules and metabolites to enter the cell [46]. It has also been reported that OmpF plays essential roles in the acid resistance of *E. coli* in the presence of arginine and lysine [47]. Therefore, we hypothesized that OmpF contributes to a certain extent to the influx of arginine and lysine across the cell envelope. The HPLC assay showed that the concentration of arginine and lysine in the wild_type strain was significantly greater than that in mutant strains, which directly proved that OmpF has a positive effect on cell permeability in *C. sakazakii*.

OmpF also plays a significant role in the drug resistance of bacteria and has been shown to interact with antibiotics such as β-lactams [48], chloramphenicol [49] and quinolone [50]. In addition, the passage of tetracycline in the magnesium-bound form across the outer membrane appeared to occur preferentially via the porin OmpF [51]. Thus, when OmpF expression decreases, it becomes more difficult for drugs such as tetracyclines, quinolones and β-lactams to enter bacteria [52]. Accordingly, it has been reported that these drugs (ciprofloxacin, trimethoprim, β-lactams, quinolone, etc.) can be used to select for multidrug-resistant mutants that exhibit decreased expression of *ompF* [53,54]. In this study, the resistance to gentamicin, ampicillin, tetracycline and ciprofloxacin of the *ompF* mutant was higher than that of WT, indicating that OmpF plays a vital role in regulating the passage of these antibiotics into *C. sakazakii*.

Interestingly, in the *ompF* complementation strain, some functions were restored; however, the level of *ompF* expression in cp*ompF* barely reached those of WT. We propose that such a result may be because the backbone pACYC184 used in cp*ompF* belongs to a low-copy-number plasmid. This phenomenon has been reported in our previous study [23]. In addition, Kim et al. reported that the expression level of the *hfq* strain cannot be filled in the *hfq* complement strain prepared using the low-copy-number pACYC184 plasmid in *Cronobacter sakazakii* ATCC 29544. Additionally, the reason may be due to the pACYC184 plasmid harboring *hfq* under a leaky inducible promoter, leading to an imbalance in Hfq production [55].

In our study, the functions of the *ompF* gene were investigated by the gene knockout technique in *C. sakazakii*. We found that this gene played a positive regulatory role in the biofilm formation, and the process is possibly mediated by LPS binding. When the *ompF* gene was knocked out, the content of LPS in the biofilm was significantly reduced in *C. sakazakii*. Furthermore, the results also showed a positive role for *ompF* in the permeability of this bacterium. This study helps to better understand the function of the *ompF* gene in *C. sakazakii* and provides a useful reference for further study of the function of the *ompF* gene in other bacteria.

## Figures and Tables

**Table 1:** The concentration of arginine and lysine in cell lysates.

Samples	Arginine (µmol·mL^−1^)	Lysine (µmol·mL^−1^)
WT	0.12 ± 0.01 ^a^	0.27 ± 0.04 ^a^
Δ*ompF*	BDL	0.11 ± 0.03 ^b^
cp*ompF*	0.09 ± 0.03 ^a^	0.27 ± 0.02 ^a^

^a,b^ Means with different superscript letters within same column are significant different (*p* < 0.05). Values are the means of triplicate samples ± SD. BDL = below detection limit.

**Table 2:** Different antibiotic resistance of *C. sakazakii* WT, Δ*ompF* and cp*ompF* strains from the size of inhibition zone.

Antibiotics	Inhibition Zone (mm)			
	WT	Δ*ompF*	cp*ompF*	25922
gentamicin	19.2 ± 0.3 ^a^	16.2 ± 0.3 ^b^	18.7 ± 0.3 ^a^	15.3
amoxicillin	19.7 ± 0.4 ^a^	17.6 ± 0.4 ^b^	19.7 ± 0.4 ^a^	17.4
kanamycin	14.7 ± 0.2 ^a^	0.0	14.7 ± 0.3 ^a^	17.5
chloramphenicol	19.3 ± 0.9 ^a^	17.8 ± 0.5 ^b^	18.3 ± 0.4 ^a^	-
tetracycline	17.2 ± 0.8 ^a^	15.1 ± 0.3 ^b^	16.4 ± 0.4 ^a^	-
ciprofloxacin	24.6 ± 0.3 ^a^	21.5 ± 0.3 ^b^	22.4 ± 0.9 ^a^	-

^a,b^ Means with different superscript letters within same row are significantly different (*p* < 0.05). Values are the means of triplicate samples ± SD.

## Supplementary Materials

The following are available online at https://www.mdpi.com/article/10.3390/microorganisms9112338/s1, Table S1: Bacterial strains and plasmids used in this study, Table S2. Primers used in this study, Table S3. Raman intensities of different wavenumbers.
