# Supplementary material for: Outer Membrane Protein F Is Involved in Biofilm Formation, Virulence and Antibiotic Resistance in Cronobacter sakazakii
Source: Microorganisms. 2021 Nov 11;9(11):2338. doi: 10.3390/microorganisms9112338 (PMC8619257; doi:10.3390/microorganisms9112338)
Supplement: Supplementary file 1 [file microorganisms-09-02338-s001.zip › microorganisms-1443012-supplementary.pdf]

microorganisms MDPI

Supplementary material

Table S1. Bacterial strains and plasmids used in this study.

Strains or plasmids	Genotype and/or characteristics	Reference or source
	Cronobacter sakazakii	
ATCC BAA-894	WT	ATCC
Δ*ompF*	Δ*ompF*::km^r	This study
cp*ompF*	Δ*ompF* with pACYC184-413	This study
	Escherichia coli	
DH5α	γ^-Φ80d*lacZ*ΔM15Δ(*lacZYA-argF*)*U169 recA1 endA1 hsdR17*(r_K^- m_K^-) *supE44 thi-1 gyrA relA1*	Kim et al.
	Plasmids	
pKD46	*oriR101 repA101*(Ts) Amp^r *araADpgam-bet-exo*	Kim et al.
pMDKUD	pMD18-T	This study
pACYC184	p15A *ori* Cm^r Tet^r	Kim et al.
pACYC413	pACYC184-*ompF*	This study

Table S2. Primers used in this study.

Gene amplified	Primers	Primer sequences (5′ to 3′)	Amplicon size (bp)	Note
		Mutant construction		
kana-F	Km^r cassette	C*GGATCC*GAGGTATGTAGGCGGTGC	26	*Bam*HI
kana-R		CGC*GTCGAC*ATATGTATCCGCTCATGAATT	30	*Sal*I
ompF5-F	Upstream of *ompF*	GG*GGTACC*GCGTTGTGCCTGTAGCC	25	*Kpn*I
ompF5-R		CG*GGATCC*TGTCTGTCTGGCATCTTTCC	28	*Bam*HI
ompF3-F	Downstream of *ompF*	CGC*GTCGAC*GAAATCACAATGGAACCTCGTC	31	*Sal*I
ompF3-R		CCC*AAGCTT*TCCCGTCTGCTGGTTTCG	27	*Hin*dIII
		Complementation		
Cp413-F	*ompF* gene sequence	CG*GGATCC*TTCTATTACGGTTTCACGG	27	*Bam*HI
Cp413-R		CGC*GTCGAC*ACGAGGTTCCATTGTGATT	28	*Sal*I

Table S3. Raman intensities of different wavenumbers.

Wave numbers	Raman intensity (a.u.)		
	WT	Δ*ompF*	cp*ompF*
852.63	49803.93[a]	12332.86[b]	22702.15[c]
1002.94	46893.56[a]	10611.55[b]	21559.08[c]
1126.76	48777.15[a]	11321.138[b]	22094.28[c]
1287.73	52595.36[a]	16261.23[b]	28445.75[c]
1451.74	51277.15[a]	13301.40[b]	24290.73[c]

[a, b, c] in the same raw with different letters indicate significant difference at the 0.01 level according to one-way ANOVA.
